# Clinical Holistic Medicine: Mental Disorders in a Holistic Perspective

**DOI:** 10.1100/tsw.2005.41

**Published:** 2005-04-12

**Authors:** Søren Ventegodt, Niels Jørgen Andersen, Shimshon Neikrug, Isack Kandel, Joav Merrick

**Affiliations:** ^1^ Nordic School of Holistic Medicine and Quality of Life Research Center, Teglgårdstræde 4-8, DK-1452 Copenhagen K, Denmark; ^2^ The Scandinavian Foundation for Holistic Medicine, Sandvika, Norway; ^3^ Norwegian School of Management, Sandvika, Norway; ^4^ Faculty of Social Science, Academic College of Judea and Samaria, Ariel, Israel; ^5^ National Institute of Child Health and Human Development, Faculty of Health Sciences, Ben Gurion University of the Negev, Beer-Sheva and Office of the Medical Director, Division for Mental Retardation, Ministry of Social Affairs, Jerusalem, Israel

**Keywords:** quality of life, QOL, philosophy, human development, holistic medicine, public health, holistic health, holistic process theory, life mission theory, group therapy, Denmark

## Abstract

From a holistic perspective, psychiatric diseases are caused by the patients unwillingness to assume responsibility for his life, existence, and personal relations. The loss of responsibility arises from the repression of the fundamental existential dimensions of the patients. Repression of love and purpose causes depersonalization (i.e., a lack of responsibility for being yourself and for the contact with others, loss of direction and purpose in life). Repression of strength in mind and emotions leads to derealization (the breakdown of the reality testing, often with mental delusions and hallucinations). The repression of joy and gender leads to devitalization (emotional emptiness, loss of joy, personal energy, sexuality, and pleasure in life).The losses of existential dimensions are invariably connected to traumas with life-denying decisions. Healing the wounds of the soul by holding and processing will lead to the recovery of the person's character, purpose of life, and existential responsibility. It can be very difficult to help a psychotic patient. The physician must first love his patient unconditionally and then fully understand the patient in order to meet and support the patient to initiate the holistic process of healing. It takes motivation and willingness to suffer on behalf of the patients in order to heal, as the existential and emotional pain of the traumas resulting in insanity is often overwhelming. We believe that most psychiatric diseases can be alleviated or cured by the loving and caring physician who masters the holistic toolbox. Further research is needed to document the effect of holistic medicine in psychiatry.

## INTRODUCTION

Genuine mental disorders are characterized by the condition medically referred to as "psychosis", a state of severe mental illness making normal function impossible for the patient. Psychosis is a difficult and much-debated concept, and over the years, various psychiatrists and schools of psychiatry have fought over the definition and delimitation of the term. Indeed, it is difficult to draw a clear line between psychosis and the normal, neurotic, and disturbed mental state that characterizes a large fraction of people in the western world. In Danish society, for example, often said to be one of the richest and most healthy communities in the world, one in every fourth person is severely mistriving[1,2]. Only every second has close friends with whom they share everything, every second has some kind of sexual problems, and only one in three are really satisfied with their job[1,2]. From the high numbers of prevalence and incidence presented by the major textbooks[3], about one in five will be treated by a psychiatrist during their lifetime and presumably many more will experience a severe life crisis.

## A GENERAL HOLISTIC THEORY OF MENTAL ILLNESS

The interesting question is whether there is a smooth transition from the normal state of consciousness into what we label as the psychotic state, or whether an actual qualitative shift occurs when people become mentally ill. Holistic medicine regards most dimensions of the mind as continuous, while conventional psychiatry insists that there is a discrete leap from the normal mental state to the psychotic state. Admittedly, one easily gets the impression that the mentally ill, hallucinatory patient has had a sharp break from reality. We have followed patients closely in and out of psychosis many times now; we have never observed such leaping in and out of the psychotic state. Instead, we find gradual shifts from severe existential pain, though degrees of escapes from the overwhelming emotions, to sheer denial and total repression of the emotionally painful content, and finally, into the state of hallucination as the ultimate escape from unbearable emotional pain.

There seems to be a general agreement that psychosis is characterized by a combination of the following:

- Derealization — breakdown of the reality testing, mental delusions, hallucinations
- Devitalization — emotional emptiness, loss of joy, personal energy, sexuality, and pleasure
- Depersonalization — lack of responsibility for being oneself and for the contact with others, loss of direction and purpose in life

Together, the breakdown in these three vital areas of human existence (corresponding to the fundamental dimensions of existence in the theory of talent[4]) constitutes a mental and existential state that prevents the patient from assuming responsibility for his or her own life and for normal functioning, which are the core characteristics of the psychotic state of being.

In our opinion, the most important single dimension of psychosis and "madness" in general is that the person disclaims responsibility for his or her own life. Accordingly, we consider psychosis a defense against the emotional and existential suffering associated with assuming responsibility. It might seem rather surprising that assuming responsibility can cause such emotional problems. In therapy, the extreme and intolerable pain of the psychotic patient reveals itself as arising from being yourself fully as a child (so vulnerable and open as you enter life) and failing completely in giving what you have to offer to the people you trusted fully and loved so unconditionally. As most children do not receive the holding they need[4,5], it is not so surprising that most people carry deep wounds in their soul. These wounds can burst open when life becomes rough. The real mystery is why some people choose to dig into this old, painful material voluntarily, seemingly with the intention to integrate what was left of being and thus heal their existence, while other people keep the mind extroverted and the machinery of the facade intact through life, thus avoiding the turmoil connected with confronting the most serious of our human traumas.

As psychosis comes in a gradual spectrum, its most common manifestation is "silent" and indistinguishable to lay people, such as the quiet young girl who confesses to her physician that her home has been equipped with surveillance cameras, only she cannot find them. It may also have very dramatic manifestations as when the patient poses a danger to himself and to others, as in classic madness. In this situation, a conflict is often building over time and the patient often chooses to be evil in order to avoid emotional pain[6].

When encountering a psychotic patient in the holistic clinic, we have to examine the following conditions to assess the nature and severity of the psychosis. For the patient's own safety, in particular, it is important to assess whether the severity of the psychosis prevents survival in a normal, everyday life. The degree of reduced functioning and the severity of the psychosis call for special precautions:

- Cognitive disturbances — Is the patient's perception impaired or distorted; for example, by hallucinations?
- Emotional disturbances — Is the basic mood lowered as in the case of depression or elevated as in the case of mania? Is the sex life affected?
- Disturbances in meaning, content, and direction of life — Is the patient realistic in respect to his or her project in life? Is the patient assuming responsibility for his or her own existence and the relations to other? Is the patient consciously choosing to be evil?

Healthy individuals are in control of their fantasy world and do not mix it with the perception of the external world, while psychotic patients tend to hallucinate and create their own perceptions, like an internal picture partially overlapping the perception of the external world. When studied extremely carefully, it can be demonstrated that everybody projects something onto other people from their own subconscious mind (like the inner man or woman when falling in love). Everybody is slightly hallucinatory, what really matters is the degree.

In some psychotic patients, the perception of reality includes a few, perceptional elements created unconsciously. The classic example of such acute psychosis is delirium tremens in alcoholics, where spiders and snakes crawl out of the walls. The cause of delirium in alcoholics is due to the brain compensating for the sedative effect of alcohol by increasing activity, including the neurotransmitter system that uses a substance called GABA. When the alcoholic suddenly drinks less than usual, brain activity becomes so high and productive that the symbols and images otherwise belonging to the dream world and the subconscious mind cannot be contained within the imagination, but are projected onto the walls and doors of the physical world.

The examination will often reveal that any patient is, to some extent, living in his or her own world, which is one way of disclaiming responsibility. A total lack of reality testing — where the patient lacks the ability to respond appropriately to the external environment and has withdrawn completely into his or her own reality — is rare. Even in the most hallucinatory state of being, most aspects of reality still are interpreted normally (there might be nonexistent spiders in the state of delirium, but they still climb the real wall and table).

The spontaneous hallucinations observed in most mentally ill patients are difficult to understand. While the patient is unwilling to take responsibility for the pain from the traumatic moments in the past, at the same time, urgent matters in the subconscious mind probably cause them. The problem manifests itself symbolically in the present: old poisonous comments become poisonous gas flowing into the house, old condemnation becomes hazardous irradiation, childhood traumas from excessive control become a sense of camera surveillance at home, the parents' unbearable criticism turns into constantly audible voices.

Another strategy for disclaiming responsibility is to depreciate the existence, value, power, and possession of your "self", of life and the world, obviating all requirements for achievement and performance. When the person is so insignificant and has so little value or knowledge and the world is so impossible — the perception observed in many depressive patients — the person no longer has any particular responsibility for his or her life. No one is committed beyond one's power.

Conventional psychiatry distinguishes between two main types of mental illness: *schizophreniform disorders*, which primarily occur in schizophrenic patients and borderline patients, and *affective mood disorders*, which occur in depressive patients, manic patients, and patients with manic-depressive disorders. The former type is generally associated with increased brain activity and can be treated with psychotropic drugs, which reduce the brain activity appropriately. By contrast, a depressive patient's brain activity is too low. This can be treated with drugs that stimulate brain activity. Anxiety is related to specific neurotransmitter systems and can be treated with drugs that suppress the activity of these systems.

Psychotropic drugs have come into wide use in our culture and at least one in five people in Denmark will receive such drugs at some point in life. There is consensus that psychotropic drugs affect the symptoms, but not the actual disorder. To heal the disease, the patients must heal his or her existence and human character[7] and, in this process, recover the clearness of mind, the spaciousness of feelings, the strength of being present in the body, the acceptance of gender and sexuality, and the acknowledgment of the essence of his wholeness and being (the soul) and in the core of this: the purpose of life[8].

Before we address the question of how the psychiatric patient can be helped by the holistic physician, we take a closer look at the holistic process of healing. It is important to notice that the holistic theory of mental illnesses presented here is derived from a general theory of loss of health, quality of life, and ability[4,5,6,7,8,9,10]. From a holistic perspective, psychiatric diseases are caused by the patient's unwillingness to assume responsibility for his or her own life, existence, and personal relations. This loss of responsibility is caused by the repression of the patient's fundamental existential dimensions, which we normally call love and purpose, strength and power, joy, gender and sexuality[4]. Repression of love and purpose leads to *depersonalization* or the lack of responsibility for being oneself and for the contact with others, as well as loss of direction and purpose in life. Repression of power and strength in mind, feelings, and body leads to *derealization* or the breakdown of the reality testing, often with mental delusions and hallucinations. The repression of joy, gender, and sexuality leads to *devitalization* or emotional emptiness, loss of joy, personal energy, sexuality, and ability to feel pleasure and happiness in life. The loss of the physical, mental, and spiritual character seems to be the price the patient has to pay for this multidimensional repression of his or her true self.

## CLINICAL HOLISTIC MEDICINE

The life mission theory[4,5,6,7,8,9,10] states that everybody has a purpose of life or huge talent. Happiness comes from living this purpose and succeeding in expressing the core talent in your life. To do this, it is important to develop as a person into what is known as the natural condition, a condition where the person knows himself and uses all his efforts to achieve what is most important for him. The holistic process theory of healing[11,12,13,14] and the related quality of life theories[15,16,17] state that the return to the natural state of being is possible whenever the person gets the resources needed for existential healing. The resources needed are "holding" in the dimensions of awareness, respect, care, acknowledgment, and acceptance with support and processing in the dimensions of feeling, understanding, and letting go of negative attitudes and beliefs. The preconditions for the holistic healing to take place are trust and the intention for the healing to take place. Existential healing is not a local healing of any tissue, but a healing of the wholeness of the person, making him much more resourceful, loving, and knowledgeable of himself and his own needs and wishes. In letting go of negative attitudes and beliefs, the person returns to a more responsible existential position and an improved quality of life. The philosophical change of the person healing is often a change towards preferring difficult problems and challenges, instead of avoiding difficulties in life[18,19,20,21,22,23,24,25]. The person who becomes happier and more resourceful often also becomes more healthy, more talented, and more able to function[26,27,28].

## DIMENSIONS OF THE MENTAL DISORDERS

A skilled psychiatrist will immediately "scan" his patient for a dozen or so different symptoms, more or less well defined. Some of them are so well defined that they can be rated on various psychometric scales, which will indicate the severity of the patient's condition: depression, mania, anxiety, psychosis (e.g., hallucinations), neuroticism, or introversion. Other dimensions can be sensed, but are difficult to quantify: the degree of delusion, somatization, grief, hypochondria, arousal level (e.g., panic), liveliness, untruthfulness, hysteria, and quality of attention or alertness.

Based on these observations, the psychiatrist can form an impression of the degree of the patient's suffering, functional capacity, the degree to which the patient assumes responsibility for his or her own life and relations, the patient's level of consciousness and insight, and finally, the severity of the disorder. The complexity of human consciousness makes it difficult to become a good holistic psychiatrist because of the numerous paths that one has to know and be able to follow as the patient enters them.

Conventional psychiatric treatment typically involves psychotropic drugs or electroconvulsive therapy (ECT). In the short run, the drugs and ECT are efficient in about half the patients. Generally, there is insufficient evidence on the long-term effect of the drugs and little scientific knowledge about any lasting effect of the often-serious temporary side effects of ECT, like discomforts and memory impairment[29]. After a search in Medline (www.pubmed.gov), it seems that the long-term side effects have not been well examined, but it also seems fair to expect that at least some side effects will last from this extremely violent treatment. Conventional psychiatric treatment seeks to alleviate the symptoms that prevent the patient from functioning and coping with life. It is generally agreed that while medication can be effective in many cases, it hardly ever leads to recovery. Many health professionals would, therefore, prefer a new psychiatric approach that deals more with the causes of the disorders. In order to adopt such an approach, we have to understand the causes of psychiatric disorders.

> **Female, aged 27 years, where the psychiatrist only prescribes medication:** Patient is dissatisfied because her psychiatrist only prescribes medication. Physiotherapy hashad little effect on her headache. Patient has had physiotherapy ten times, and there is no reason to continue. I recommend her to read books about people who have had the same experience and have solved their problems. Perhaps the librarian can help her. She has to be honest with her psychiatrist and verbalise her discontent.

As physicians, we must to be careful not to destroy each other's work. When the psychiatrist has put the patient on medication, in principle, we should not interfere with his field of work. However, since this patient is dissatisfied with the psychiatrist and seeks help from a holistic physician, she obviously feels a need to get help and support to confront the biomedical paradigm[30,31]. We believe that reading books is important, as they can provide words with which to think. They will also make it easier for the patient to communicate with the psychiatrist. In this case, the helper is the librarian.

## BIOMEDICAL VS. HOLISTIC PERSPECTIVES OF THE MENTAL DISORDERS

In a conventional (biomedical) psychiatric perspective, mental disorders are caused by certain disturbances in brain activity, considered to be genetically controlled. Depression and schizophrenia are assumed to be hereditary, although there is insufficient evidence of any genetic causes. Studies with identical twins who grew up away from each other showed that in 25–50% of the twin pairs, both twins were schizophrenic (the concordance)[32,33,34,35,36]. This is generally considered to support the hypothesis that schizophrenia has a genetic cause, but in our opinion, it confirms the belief that factors other than genetics determine whether the disorder develops. There is, of course, some genetically determined vulnerability that might differ from individual to individual, but the genetic patterns have never been identified with any certainty, so this is still speculative. When we take mentally ill or disturbed patients into deep regressive therapy, many patients reveal traumatic episodes going all the way back to the womb. It is very likely that identical twins, being genetically identical, are in closer mental contact in the womb than nonidentical twins, making them share the content of their early consciousness, also the traumatic content, to a higher extent. Such considerations seem to favor the hypothesis of early psychosocial factors causing schizophrenia and weaken the evidence for the genetic hypothesis.

From our holistic perspective, mental disorders are generally not caused by genetics. People may be genetically predisposed, but by traumas (emotionally difficult situations often occurring early in the individual's life that lead to negative decisions denying life, self, and reality). The decisions lie as deep structures in the conscious and subconscious mind and compromise the patient's relations with himself, his inner life, and the outside world. The inner conflicts are manifested as suffering and reduced capacity in relation to mental and social functioning, love, sex, or work.

The traumas in the mentally ill patient often involve severe emotional pain, leading to dramatic and destructive statements such as: "I am outside", "Nobody likes me", "I am nothing but trouble", "I am crazy", "I am dead", "You are dead", "It is unreal", "It is not now". Often, these statements include a directly social hereditary element, for instance, if the patient's mother has had a mental disorder and experiences with her led to conclusions such as: "She is mentally ill," and if one attempts to excuse the mother's illness: "I am mentally ill," etc.

## HOLISTIC HEALING INDUCES RECOVERY OF CHARACTER, PURPOSE, AND RESPONSIBILITY

Recovery is known to happen in one out of four patients, even in the most severe psychiatric cases. The recovery literature shows several kinds of recovery from schizophrenia[32], the most interesting being full recovery happening in one study in 13.7% of the patients after 5 years[33] and in about 25% of the patients long term[34,35] in the western countries and, quite surprisingly, much more often in the third world[36]. Since third-world countries are mostly without a developed, biomedical psychiatry, this may indicate that many of the therapeutic procedures that seem beneficial in the short run (month) might actually be contratherapeutic with a perspective of years. It is important to cooperate with this spontaneous recovery process and to enhance it if and when possible. This is the mission of the holistic approach to the psychiatric illness.

According to the holistic medicine perspective, the major mental disorders are caused by traumas with painful emotional content and life-denying decisions. Therefore, the causal cure consists of helping the patient to heal his or her existence by the integration of the old traumas. Existential healing is induced by applying the obligatory steps of holistic process of healing on the mental diseases[13]:

- Make the patients become aware of what lies behind the symptoms they display.
- Let them sink into the feeling until they understand what it is about.
- Help them apply words to the feelings and support them in letting go of all decisions that make themselves or their lives less good and real.

In practice, the process is complicated. The greater the old emotional pain, the stronger the patient's mental defenses against encountering the pain now and the more support and holding (attention, respect, care, acceptance, and acknowledgment) are required to get the patient through the trauma. In principle, holistic medicine can help any psychiatric patient who is willing to assume responsibility for his or her own life, provided that there is sufficient support and that the holistic physician fully understands the patient, his/her situation, and his/her state of being. The latter is absolutely crucial. A patient who does not experience being seen and met will not be able to show any trust. Without trust, the patient will not allow the physician to give him/her holding and support.

To understand mentally ill people in sufficient depth, the therapist must possess great personal insight and acknowledge the corresponding problems in his own life, naturally on a smaller scale. In our opinion, we are all tarred with the same brush regardless of the nature and severity of the particular human problem. The purpose of conventional, biomedical, psychiatric treatment of psychosis is to make the symptoms of the psychosis disappear, while the purpose of holistic treatment of mental disorders is to eliminate their cause and, in that way, help the patient return fully to life, health, and ability. The essence of holistic treatment of mental disorders is to help the patient to recommence and take full responsibility for his or her life. As they disclaim their responsibility under extreme pressure, patients frequently have to relive difficult events and temporarily experience increased aggravation and suffering during the sessions in order to recover. This constitutes a distinct difference between biomedical and holistic psychiatry. The former approach does not allow the patient to suffer, because suffering is unnecessary, while the latter approach allows suffering if it helps the patient to move on. In addition, existential pain is actually an important element of life of which the patient should not be deprived without the most careful of thoughts.

In the holistic clinic, it is rational to apply the conventional (biomedical) psychiatric diagnoses to some extent. This allows for consensus among professionals when cooperating in helping the patient and when doing research. However, the holistic physician has to supplement the traditional diagnosis with a description of the dimensions that are relevant for the holistic therapy. For example: What resources, internal and external, does the patient possess? What is the patient's reality? Is the patient, for example, an institutionalized, experienced user of psychiatry, with little motivation for a major change, or is it a new patient with no experience with the established psychiatric system? Is there an insatiable appetite for learning and a will to recover?

The latter questions are very important because patients can survive in the psychiatric system with a much lower level of responsibility than is required to survive in the real world. Patients who have become accustomed to being hospitalized know that it is acceptable for them to disclaim responsibility, unlike patients who lack that experience. In a way, the biomedical, psychiatric system of today inadvertently rewards patients who disclaim responsibility, which is most unfortunate.

In the field of holistic psychiatry, the physician's kindness, generosity, and emotional capacity are the primary tools for helping the patient. The physician's love of his patient is the patient's primary resource and the ethical standard of the holistic physician is also an important prognostic factor. The physician's good intention restores confidence and provides an opening. If a therapist has a patient that he cannot accommodate, it is absolutely essential to say so immediately and refer the patient to someone who can. Incidentally, kindness is neither sympathy nor empathy, but rather the willingness to give something to the patient without receiving anything in return, a generous quality closely related to the love we share with our relatives.

## SUICIDAL THOUGHTS

Naturally, suicidal thoughts are a central issue since a patient with very low self-esteem may have a spontaneous death wish in order not to be in the way or cause trouble for other people. The way we see it, a death wish is actually social. We all, deep down, need to feel useful and if we are not of value to anybody, we do not wish to live as a burden.

Specific plans to commit suicide means that the patient has to be treated in cooperation with an experienced psychiatrist. If the situation is clearly life threatening, there is no alternative to admission for treatment, by force if necessary. As force will almost invariably inflict new traumas on the patient and therefore cause a setback in the patient's development, force should only be applied in extreme cases. If the physician succeeds in making the patient let go of the decisions that are the cause of low self-esteem and the death wish, a crisis can sometimes be avoided, but it is important to ensure that the patient's condition subsequently remains stable. With this said, it really is a difficult ethical (and classical philosophical) question: Are we allowed to compromise the patient's autonomy to save his life? What is more important: the life *or* the survival of the patient? If we have to choose, most people will say that surviving with no living is pointless. Of course, living is not possible without survival either.

## DISCUSSION

Patients with acute psychosis, who do not pose any danger to themselves or others, are highly susceptible to contact and care founded on profound empathy and endless patience. Acute psychosis is a common condition in connection with severe traumas and may also be provoked by recreational substances such as LSD, ecstasy, or cannabis. Follow-up should include a brief series of conversations. If the patient remains in a psychotic state, a psychiatric specialist can provide help by means of appropriate, small doses of antipsychotic medication.

Unipolar depression, including major depression and minor depression, can often be treated with holistic medicine. A therapeutic course lasting 6 months should be expected in major depression. Antidepressants can often remove the symptoms in a couple of months, but the tendency towards depression will usually persist for the rest of the patient's life. Once the holistic treatment has been completed successfully and the patient has learned from the experience, there is justified hope that the depression is gone forever.

In our experience, the bipolar disorder is difficult to treat because this type of patient tends to shift rapidly from one mental state to the next to avoid contact with the underlying existential pain. In young people who have not been admitted for psychiatric treatment, but have a tendency to become psychotic, holistically oriented conversational therapy appears to be effective in preventing mental illness, but more research is required to confirm this. Schizophreniform psychosis (schizophrenia, borderline psychosis, and similar disorders) can be treated if the therapist understands and feels great kindness towards the patient. A long, therapeutic course should be anticipated because psychosis usually reveals a hidden flaw in the patient's character in respect to responsibility.

In elderly psychotic patients or patients with a history of repeated hospitalization in a psychiatric ward, treatment may be extremely difficult and require substantial resources. For example, in the case of the patient's resources (for example, because of the patient adapting to a life as mentally ill with all the privileges of not being responsible), holistic treatment without psychotropic medication is deemed impracticable. A mentally ill patient often undergoes thorough assessment and a detailed treatment and development plan prior to receiving holistic therapy, which is frequently provided in close cooperation with a psychodynamically oriented psychiatrist. Now and again, we let the specialist do the initial work with the patient, particularly in patients who require many resources, then we step in as a coach for personal development when the patient has been stabilized and has gained access to his own resources. People with development perspective are generally more susceptible to holistic treatment than people who are ignorant of the notion of personal development.

From a holistic perspective, most psychiatric diseases are caused by the patient's unwillingness to assume responsibility for his life, existence, and personal relations. The loss of responsibility arises (according to the holistic theory of mental illnesses) from the patient's repression of the fundamental existential dimensions (called love, strength, and joy in the theory of talent). Repression of love and purpose leads to depersonalization (lack of responsibility for being oneself and for the contact with others, loss of direction and purpose in life). Repression of strength in mind and emotions leads to derealization (the breakdown of the reality testing, often with mental delusions and hallucinations). The repression of joy and gender leads to devitalization (emotional emptiness and loss of joy, personal energy, sexuality, and pleasure in life).

The loss of existential dimensions is invariably connected to traumas with life-denying decisions. Healing the wounds of the soul by holding and processing in accordance with the holistic process theory of healing will lead to the recovery of the person's character, purpose of life, and existential responsibility. It can be very difficult to help a psychotic patient. The physician must first love his patient unconditionally and then fully understand the patient and his/her state of being. Only then can he meet and support the patient and initiate the holistic process of healing. It takes a lot of motivation and willingness to suffer on behalf of the patient so that he can heal, as the existential and emotional pain of the traumas giving insanity is often overwhelming. We believe that most psychiatric diseases can be alleviated or cured by the loving and caring physician who masters the holistic toolbox. Further research is needed to document the effect of holistic medicine in psychiatry.

## ACKNOWLEDGMENTS

This study was supported by grants from IMK Almene Fond. The quality of life research was approved by the Copenhagen Scientific Ethical Committee under number (KF)V.100.2123/91.

## BIOSKETCHES

**Søren Ventegodt, MD,** is the director of the Nordic School of Holistic Health and Quality of Life Research Center in Copenhagen, Denmark. He is also responsible for a Clinical Research Clinic for Holistic Medicine in Copenhagen and is a popular speaker throughout Scandinavia. He has published numerous scientific or popular articles and a number of books on holistic medicine, quality of life, and quality of working life. His most important scientific contributions are the comprehensive SEQOL questionnaire, the very short QoL5 questionnaire, the integrated QOL theory, the holistic process theory, the life mission theory, and the ongoing Danish Quality of Life Research Survey, 1991–94 in cooperation with the University Hospital of Copenhagen and the late professor of pediatrics, Bengt Zachau-Christiansen, MD, PhD. E-mail: ventegodt@livskvalitet.org. Website: www.livskvalitet.org/

**Niels Jørgen Andersen, MSc,** professor, Department of Innovation and Economic Organization, Norwegian School of Management. This department conducts research and provides teaching in central topics related to innovation, business development, management of global companies, business history, and economic organization. Mr. Andersen's research activities within the Department are related to four core subjects within the discipline: business history, cooperative organizations, business development and entrepreneurship, and finally, studies of industries with a special focus on the electricity industry. He is also the dynamic chairman of the nonprofit organization, Stiftelsen Holistisk Medisin Scandinavia, which aims to support the scientific development, research, and documentation of complementary and holistic medicine in Scandinavia. E-mail: niels.j.andersen@bi.no. Website: http://www.bi.no/users/fgl93013

**Shimshon Neikrug, PhD,** is a senior lecturer in the Department of Social Work at the College of Judea and Samaria, Ariel, Israel, where he is the director of community studies. He has lectured in Social Work at Tel Aviv University and Bar-Ilan University, as well as serving as the director of the research program in the Bar-Ilan Brookdale Program. Dr. Neikrug has served as consultant to community projects in both the Palestinian Arab sector and Jewish sectors. He is the founder and chair of Yakir-Association for the Third Age. In that capacity, he has directed research projects on quality of life for both private- and public-sector organizations. His present research is on the development of informal support networks for families of children with special needs. E-mail: neikrug@bezeqint.net, Website: http://www.yosh.ac.il/socialwork/Faculty.asp?n=Neikrug_S

**Isack Kandel**, **MA, PhD,** is senior lecturer at the Faculty of Social Sciences, Department of Behavioral Sciences, the Academic College of Judea and Samaria, Ariel. During the period 1985–93, he served as the director of the Division for Mental Retardation, Ministry of Social Affairs, Jerusalem, Israel. E-mail: Kandeli@aquanet.co.il

**Joav Merrick, MD, DMSc,** is professor of child health and human development affiliated with the Zusman Child Development Center, Division of Pediatrics and Community Health at the Ben Gurion University, Beer-Sheva, Israel; the medical director of the Division for Mental Retardation, Ministry of Social Affairs, Jerusalem; and founder and director of the National Institute of Child Health and Human Development, Faculty of Health Sciences, Ben Gurion University of the Negev. He has numerous publications in the field of child health and human development, rehabilitation, intellectual disability, disability, health, welfare, abuse, advocacy, quality of life and prevention. Dr. Merrick received the Peter Sabroe Child Award for outstanding work on behalf of Danish Children in 1985 and the International LEGO-Prize ("The Children's Nobel Prize") for an extraordinary contribution towards improvement in child welfare and well being in 1987. E-mail: jmerrick@internet-zahav.net. Website: www.nichd-israel.com
